# Monoclonal antibody potentiating gonadotropin activity *in vitro* and *in vivo* in male and female rats and in ewes

**DOI:** 10.1530/REP-25-0212

**Published:** 2025-09-15

**Authors:** Elodie Kara, Jérémy Decourtye, Laurence Dupuy, Sophie Casteret, Philippe Bouchard, René Frydman, Marie-Christine Maurel

**Affiliations:** ^1^ Igyxos Biotherapeutics, Centre INRAE Val de Loire, Nouzilly, France; ^2^ Hôpital Foch, Service de Gynécologie Obstétrique, Suresnes, France

**Keywords:** antibody, fertility, follicle-stimulating hormone, luteinizing hormone, chorionic gonadotropin, modulation, potentiation, spermatogenesis, ovulation

## Abstract

**In brief:**

Treatment of female and male infertility currently depends on repeated injections of gonadotropins, which can be burdensome for patients and do not always provide successful outcomes. Based on different animal models, CF12 mAb potentiates the effect of both exogenous and endogenous gonadotropins in females and males, suggesting its potential to improve outcomes and reduce the burden of infertility treatments.

**Abstract:**

Follicle-stimulating hormone (FSH) and luteinizing hormone (LH) are key for folliculogenesis and spermatogenesis and play a role, together with chorionic gonadotropin (CG), in fertility treatment. In ewes and goats treated with equine CG (eCG), some females secreted antibodies directed against eCG that potentiate its activity and are associated with a higher birth rate. Starting from this observation, we developed CF12, an anti-gonadotropin monoclonal antibody (mAb), to potentiate FSH and LH/CG bioactivity. Following *in vitro* studies that explored CF12 mAb’s impact on FSH, LH, and CG potency on cyclic AMP (cAMP) production in HEK293 cells expressing respective receptors, we proceeded to examine the effects *in vivo*. We established in immature female and male rats that the effect of CF12 mAb is dose dependent. In an adult male azoospermic rat model, CF12 mAb in combination with gonadotropins completely restored spermatogenesis within one spermatogenesis cycle, whereas treatment with gonadotropin only failed to do so. Ovulation was induced by CF12 mAb alone in all treated primiparous ewes (*n* = 7; 100%), demonstrating its potentiating effect on endogenous hormones (by comparison, treatment with porcine FSH (pFSH) induced just four ovulations in 11 treated ewes; 36%). CF12 mAb also induced better progesterone secretion compared to the pFSH group. In conclusion, CF12 mAb potentiates the effect of both exogenous and endogenous gonadotropins and could be used to improve the outcome of fertility treatments. The development of a humanized variant of CF12 mAb would be of utmost interest in offering patients an alternative option for the treatment of infertility in both men and women.

## Introduction

Gonadotropins are essential to reproduction in all vertebrates, including humans. Luteinizing hormone (LH) and follicle-stimulating hormone (FSH) are present in all species, whereas evolutionarily younger chorionic gonadotropin (CG) is only found in equines and some primate species (Carter 2022). While FSH binds to its own FSH receptor (FSH-R), LH and CG share the same LH/CG receptor (LH/CG-R) (Casarini *et al.* 2021). In females, FSH stimulates immature ovarian follicles, whereas LH triggers follicular growth and ovulation, corpus luteum development, and progesterone secretion to support pregnancy; CG helps maintain the corpus luteum and early pregnancy. In males, LH enhances testosterone production by interstitial Leydig cells in the testes, whereas FSH acts on Sertoli cells to support spermatogenesis (Oduwole *et al.* 2021).

Gonadotropins are widely used to manipulate reproductive functions both in humans as part of assisted reproductive technologies (ART) (Lunenfeld *et al.* 2019) and in animals for estrus, ovulation synchronization, and artificial insemination (Habeeb & Anne Kutzler 2021). Specifically, equine CG (eCG), which binds only to LH receptor in equines but exhibits dual FSH- and LH-like activity in other species, is used to induce ovulation in livestock such as ewes, goats, cows, and sows (Roy *et al.* 1999*b*, Cline *et al.* 2001, Brito *et al.* 2024, Sales *et al.* 2024). However, a limitation to the use of eCG in this setting has arisen: its glycosylated structure renders eCG highly immunogenic in heterologous species. As a consequence, 60% of ewes and goats develop anti-eCG antibodies (Ab), resulting in a decreased and delayed response to subsequent eCG treatment, which directly correlates to lower birth rates at the individual level (Baril *et al.* 1993, Roy *et al.* 1999*a*,*b*, Maurel *et al.* 2003). Similar humoral immune responses targeting gonadotropins have been described in felids receiving a combined treatment with equine and human CG (hCG) (Swanson *et al.* 1995) and in men treated for hypogonadism with hCG (Ogura *et al.* 2003).

The anti-eCG Ab immune response developed by ewes and goats exhibits a high inter-individual variability and affects FSH bioactivity more than LH (Kara *et al.* 2019). Interestingly, these anti-eCG Abs exhibit different types of modulating activity: whereas most females develop anti-eCG Abs that decrease fertility, some females develop anti-eCG Abs resulting in enhanced fertility, reaching a 100% birth rate, sometimes with hyperprolificity (Kara *et al.* 2019). Enhancing anti-eCG Abs collected from goat plasma were purified and their action in combination with eCG on the FSH-R downstream signaling studied *in vitro*; compared to eCG alone, the enhancing complexes activate protein kinase A and beta-arrestin pathways and increase extracellular signal-regulated kinase phosphorylation in HEK293 cells expressing the FSH-R (Wehbi *et al.* 2010).

These findings prompted us to identify a monoclonal anti-human-FSH enhancing mAb and investigate its potentiating activity on folliculogenesis in females and spermatogenesis in males. Here, we describe the development of such a mAb, CF12, and a series of *in vitro* and *in vivo* experiments to investigate its ability to potentiate gonadotropins in male and female animal models. It is anticipated that these early proof-of-concept investigations will inform the future development of anti-gonadotropin potentiating antibodies for use in the treatment of infertility.

## Materials and methods

Here we describe a series of studies undertaken to demonstrate the potentiating effect of CF12 mAb on hormone activity and reproductive function, initially *in vitro* and then *in vivo* in both male and female animal models. These included immature rat models, mature male rats, and primiparous ewes. All animal procedures were conducted at Unité Expérimentale de Physiologie Animale de l’Orfrasière (UEPAO, https://doi.org/10.15454/1.5573896321728955E12, France). Protocols were carried out according to EU guidelines (directive 2010/63/EU) on the protection of animals used for scientific purposes, evaluated by the ethics committee on animal experimentation of Région Centre-Val de Loire (France), and validated by the French Ministry of Research. Details of the development of CF12 mAb can be found in the Supporting Information (see section on Supplementary materials given at the end of the article).

### Materials

All hormones used are commercially available. Recombinant human FSH (rhFSH, Gonal-f®, Cat# 3400936348110) and recombinant human LH (rhLH, Luveris®, Cat# 3400935496669) were from Merck Serono (Germany). Extracted hCG (Gonadotrophine Chorionique ENDO 5000 UI (ENDO5000), Cat# 3400930229392) was from IBSA Pharma (France). Menopur®, a human menopausal gonadotropin (hMG, Cat# 3400935681614), and Firmagon® (degarelix, Cat# 3400939432687), the gonadotropin-releasing hormone (GnRH) antagonist, were from Ferring Pharmaceuticals (France). Highly purified porcine FSH (pFSH, batch 10D22) was from Jean-François Beckers’ laboratory (University of Liège, Belgium).

### Development of CF12, an anti-rhFSH mAb

A mouse was immunized with rhFSH. Ab-producing clones were obtained after the fusion of spleen cells of the immunized mouse with Sp2/O cells. The activity of antibodies secreted by the generated clones was compared based on *in vitro* FSH bioassays using bovine granulosa cells prepared from ovaries collected from a local slaughterhouse or HEK293 cells expressing FSH-R, and on an *in vitro* LH bioassay using mLTC-1 cells. The clone secreting the antibody with the best potentiating effect on cAMP secretion went through two limiting dilutions, and a second round of *in vitro* testing was performed on these new clones. Clone CF12 showed the best potentiating activity and was selected as the lead candidate. A working cell bank was generated from this selected clone and frozen.

### SDS-PAGE

The CF12 mAb sample was prepared in reducing buffer and separated by SDS-PAGE on 4–15% precast polyacrylamide gels that were stained with Coomassie blue.

### Size-exclusion chromatography (SEC)

SEC analyses were performed using an HPLC system with a Superose 6 10/300 GL column. The mobile phase was phosphate-buffered saline (PBS) at 0.5 mL/min flow rate. Chromatograms were obtained by monitoring the absorbance at 280 nm.

### Enzyme-linked immunosorbent assay (ELISA)

ELISA 96-well Immulon 2HB plates (Nunc, Fisher Scientific, France) were coated for 1 h at 37°C, followed by 4°C overnight (ON), with 100 μL of rhFSH, hCG, or rhLH diluted at 10 μg/mL in PBS pH 7.4. Plates were washed five times with PBS and wells were blocked with 100 μL of PBS-bovine serum albumin (BSA) 1% for 1 h at 37°C. CF12 mAb serially diluted in PBS–BSA 0.2% was added in duplicate to the wells and incubated for 2 h at 37°C. Plates were then washed five times with PBS, and 100 μL of Anti-IgM conjugated to horseradish peroxidase diluted to 1/2,500 were added to each well and incubated for 1 h at 37°C. Plates were washed five times with PBS, and 100 μL of tetramethylbenzidine (TMB) substrate were added to each well. Reactions were stopped by adding 50 μL per well of 1M sulfuric acid. Absorbance was measured at 450 nm using an ELISA plate reader.

### Activity of CF12 mAb *in vitro* on gonadotropins

In order to establish the potentiating activity of CF12 mAb on FSH *in vitro*, HEK293 hFSH-R GloSensor cells overexpressing human FSH-R and cyclic adenosine monophosphate (cAMP)-responsive biosensor GloSensor™ (Promega, France), previously described by Maya Haj Hassan and her colleagues (Haj Hassan *et al.* 2015), were provided by Eric Reiter (INRAE Val de Loire, France). They were cultured at 37°C, 5% CO_2_, in a humidified atmosphere, in minimal essential medium (MEM) with 10% heat-inactivated fetal bovine serum (FBS), 100 IU/mL penicillin, 100 μg/mL streptomycin, 0.4 μg/mL geneticin, and 0.4 μg/mL hygromycin. Cells were cultured ON in white 96-well plates with 80,000 cells per well. Cell culture media were removed and replaced by 100 μL MEM containing 4 μL of GloSensor cAMP reagent, then incubated for 2 h at room temperature.

Luminescence was recorded using a POLARstar Omega plate reader (BMG Labtech, France) before stimulation. Cells were then stimulated with 10 μL of either increasing concentrations of rhFSH alone or pre-incubated with a fixed dose of CF12 mAb (10 μg/mL), or with 10 μg/mL of CF12 mAb alone, prepared and incubated in PCR mixing blocks at 37°C for 20 min. Immediately after stimulation, luminescence was recorded over 60 min. The maximal cAMP level was extrapolated and dose–response curves were generated.

In parallel, male mouse Leydig tumor cells (mLTC-1) expressing LH/CG-R were purchased from the American Tissue and Cell Collection (ATCC, LGC Standards, France) and cultured at 37°C, 5% CO_2_, in a humidified atmosphere, in RPMI 1640 supplemented with 10% heat-inactivated FBS, 50 μg/mL gentamicin, 10 IU/mL penicillin, and 10 μg/mL streptomycin. Cells were split and seeded at 80,000 cells per well in white 96-well plates. Before stimulation, cells were starved for 2 h in 30 μL PBS containing 1 mM HEPES at 37°C, 5% CO_2_. Different dilutions of rhLH or hCG with a fixed dose of CF12 mAb (10 μg/mL) were pre-mixed in PCR mixing blocks and incubated at 37°C for 20 min. The mixes (15 μL) were then added to the cells and incubated for 90 min at 37°C, 5% CO_2_. At the end of the stimulation, cAMP levels were quantified using HitHunter® cAMP assay for Biologics (DiscoverX, Eurofins, France), and dose–response curves were generated. Similarly, mLTC-1 cells were seeded at 20,000 cells/well in 96-well plates, starved 18 h later in 100 μL RPMI 1640/25 mM HEPES at 37°C, 5% CO_2_ for 2 h, and stimulated with different concentrations of hCG pre-incubated with 10 μg/mL CF12 mAb at 37°C for 20 min. After 2 h of stimulation at 37°C, 5% CO_2_, progesterone levels were quantified using an ELISA method described in Canepa *et al.* (2008). Briefly, 150 μL of goat anti-mouse IgG antibody (Uptima, Interchim, France) was coated at 0.2 μg per well of a 96-well Immuno Maxisorp plate (Thermo Scientific Nunc, France) in 50 mM carbonate/bicarbonate buffer pH 9.6 at 4°C ON. After three washes with 450 μL of wash buffer (25 mM Tris, 37 mM NaCl, 0.5 mM MgCl_2_, 0.1 g/L NaN_3_, pH 7.5, 0.05% v/v Tween), plates were saturated with 200 μL of dilution buffer (100 mM Tris, 150 mM NaCl, 2 mM MgCl_2_, 0.5 g/L NaN_3_, pH 7.5, 5 g/L BSA). Plates were dried and stored at −20°C until use. A mouse anti-progesterone mAb was added at 0.9 ng/well in dilution buffer. Ten μL of sample was added to the wells and incubated at 4°C ON. The following day, 50 μL of progesterone-11α-hemisuccinate-alkaline phosphatase diluted 1/60,000 was added to each well, and the plate was incubated for 1 h at room temperature. After washing, 150 μL of p-nitrophenyl phosphate 1 mg/mL in 100 mM diethanolamine, 5 mM MgCl_2_, pH 9.8 was added to each well. The plate was incubated 2 h at 37°C and read at 405 nm.

### Activity of CF12 mAb *in vivo* in immature rats

We used the Steelman and Pohley bioassay (Steelman & Pohley 1953) to further understand the *in vivo* action of CF12 mAb in immature (26-day-old) female Wistar Han rats (Charles River Laboratories, France). The rats were injected with 3.5 IU hCG + 0.5 IU rhFSH or 3.5 IU hCG + 0.5 IU rhFSH + CF12 mAb subcutaneously, twice a day, for 3 days. On the fourth day, animals were sacrificed, and the ovaries were dissected and weighed. Ovarian weight was normalized per 100 g of body weight. Concentrations from 0.005 to 4 μg/injection of CF12 mAb were tested.

The analogous seminal vesicle weight gain assay described by Van Hell and his colleagues (Van Hell *et al.* 1964) was conducted with immature (25-day-old) male Wistar Han rats. Rats were treated with either 1.5 IU hCG only or 1.5 IU hCG + CF12 mAb by subcutaneous injection once a day for 4 days. On the fifth day, animals were sacrificed, and seminal vesicles were dissected, weighed, and expressed per 100 g of body weight. Concentrations from 0.005 to 4 μg/injection of CF12 mAb were tested.

### Adult azoospermic rat model: treatment and analysis

Our next step was to assess the potentiating effect of CF12 mAb in 12-week-old male Wistar Han rats. The rats were acclimated for 1 week before entering the protocol and then injected with 2 mg/kg of GnRH antagonist on day 1 and 6 weeks later (D42) to inhibit spermatogenesis. After 7 weeks, rats were randomized (four per group), and treatments were started.

Two groups were treated with hormones only: 2.5 IU/kg hCG twice a week (Tuesday and Friday) and 25 IU/kg hMG 5 days a week (Monday to Friday) (Hormones X1), or 5 IU/kg hCG twice a week and 50 IU/kg hMG 5 days a week (Hormones X2). In the CF12 mAb group, CF12 mAb was added to the hormonal treatment and injected three times a week (Monday, Wednesday, and Friday) at 20 μg/kg of body weight. The control group was treated with saline. Injections were performed once a day, subcutaneously. The experimental design is summarized in Supplementary Fig. 2.

After 8 weeks of treatment with hormones +/− CF12 mAb, animals were sacrificed. Blood samples were collected post-mortem in BD Vacutainer® heparin tubes (Dutscher, France) and centrifuged for 20 min at 2,500 *g*. The plasma was aliquoted and stored at −20°C until analysis. Seminal vesicles, testes, and epididymides were dissected, weighed, and processed for further analysis.

To enumerate sperm in the testes, the testes were ground in Ham’s F12 medium with 1% sodium pyruvate and 1% HEPES and sonicated. The number of spermatid heads was counted under a microscope using a Malassez hemocytometer. Similarly, the sperm count in the epididymides was performed by cutting the epididymides into thin sections and incubating for at least 4 h at room temperature in Ham’s F12 medium with 1% sodium pyruvate and 1% HEPES. The preparations were then recovered, filtered, and the spermatozoa were counted under a microscope using a Malassez hemocytometer in the same way as the testes.

### Effect of CF12 mAb on ovulation in ewes *in vivo*

We investigated the ability of CF12 mAb to act on endogenous FSH and induce ovulation in an adult female model: ewes. As it is known that CF12 mAb potentiates ovine FSH *in vitro* (see Supporting Information Fig. 2), we treated primiparous ewes with CF12 mAb alone and compared its efficacy with pFSH treatment. Primiparous Ile de France ewes were kept indoors and fed corn silage and concentrate ration twice a day. Water was available *ad libitum*. The experiments were conducted in January and March.

Estrous cycles were synchronized using a vaginal sponge of progestagen (Chronogest® CR, 20 mg fluorogestone acetate, Intervet, France) for 14 days. Eighteen ewes were randomized to two groups: pFSH alone and CF12 mAb alone. Group 1 ewes (*n* = 11) were treated with pFSH, in which females were injected intramuscularly twice the day before vaginal sponge removal: 100 μg in the morning and 90 μg 12 h after the first dose. Group 2 ewes (*n* = 7) that were treated with CF12 mAb received two intramuscular injections 24 h apart: 1 mg of CF12 mAb the day before sponge removal and 1 mg on the day of sponge removal.

The presence and the number of corpora lutea were determined by laparoscopy, performed 8 days after vaginal sponge removal, using the Cognié *et al.* method (Cognie *et al.* 2007). Animals were deprived of food the day before scanning and anesthetized with ketamine (Imalgène® 2.5 mL IV and 7.5 mL IM, Boehringer Ingelheim Animal Health, France) on the morning of the laparoscopy, which lasted ≤5 min.

Blood samples were taken from each ewe between days 1 and 19 post vaginal sponge removal and used to quantify progesterone by an ELISA method (Canepa *et al.* 2008).

The experimental design is summarized in Supplementary Fig. 3.

### Statistical analysis

All data were analyzed for normality using the Shapiro–Wilk test. *In vitro* experiments were repeated at least three times, and data were compared using a two-way ANOVA. *In vivo* experiments were performed on five animals per group for the Steelman and Pohley and Van Hell bioassays, and on groups of four for adult rat experiments, and were analyzed using a non-parametric one-way ANOVA (Kruskal–Wallis test) followed by Dunn’s post-test. Progesterone secretion from ewes was compared between groups using a two-way ANOVA followed by a Tukey’s post-test. Statistical analysis was performed using GraphPad Prism 10 (GraphPad Software, Inc., USA). Data were expressed as mean ± standard error of the mean (SEM). Differences were considered significant when *P* < 0.05.

## Results

### Characterization of the CF12 mAb murine anti-gonadotropin antibody

**Figure 1:**
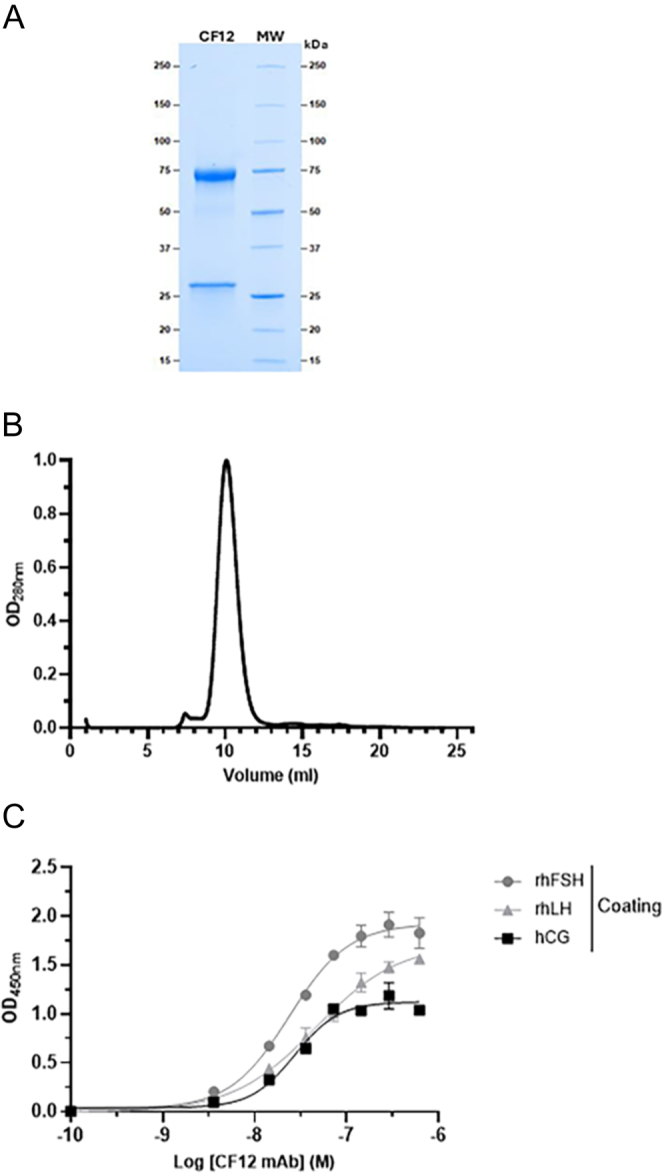
Purity and affinity for rhFSH, rhLH, and hCG of CF12 mAb. (A) Coomassie blue-stained SDS-PAGE analysis of CF12 mAb confirmed the isotype with bands at 75 and 25 kDa. (B) SEC analysis with a clear peak shows that the preparation is homogenous and well purified, with no protein contamination. (C) ELISA showing high-affinity binding to rhFSH (KD: 23.9 ± 4.8 nM), rhLH (KD: 75.2 ± 9.0 nM), and hCG (KD: 27.8 ± 3.5 nM).

The isotype of the murine CF12 mAb was established as being an IgM and confirmed by sodium dodecyl sulfate–polyacrylamide gel electrophoresis (SDS-PAGE) analysis, exhibiting two bands at 75 and 25 kDa (Fig. 1A). Size exclusion chromatography (SEC) analysis demonstrated that the CF12 mAb preparation was homogenous, well purified, and not contaminated by other proteins (Fig. 1B). ELISA showed that CF12 mAb binds with high affinity to rhFSH, rhLH, and hCG (Fig. 1C).

### Demonstration of the hormone-potentiating activity of CF12 mAb *in vitro*

**Figure 2:**
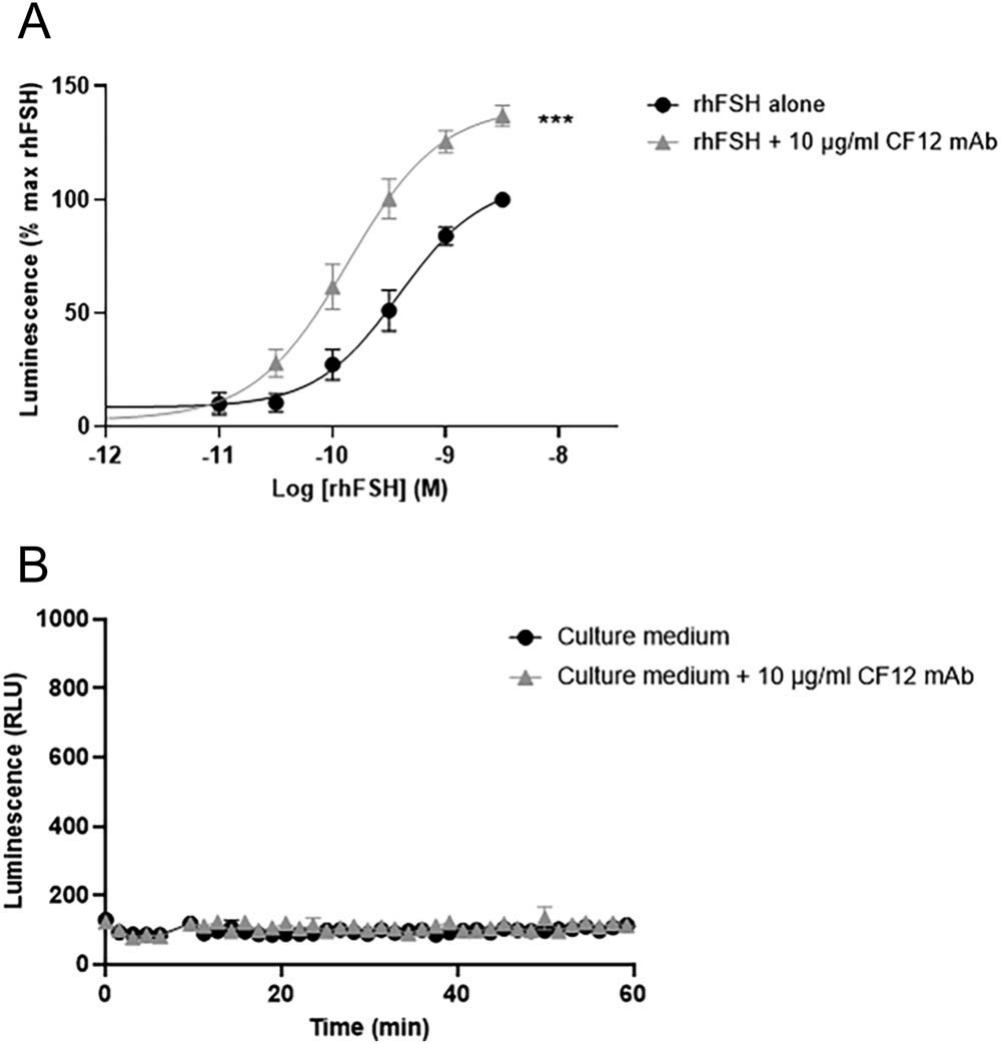
Increase in cAMP activity in HEK293 cells overexpressing hFSH-R and the GloSensor® stimulated with increasing doses of rhFSH ± 10 μg/mL CF12 mAb (A) compared with 10 μg/mL CF12 mAb only (B). (A) The maximal luminescence produced was fitted to a log (agonist) versus response curve as mean ± SEM. *n* = 8 independent experiments done in duplicate. Results are expressed as % of maximal rhFSH effect. The addition of CF12 mAb to rhFSH significantly increased the cAMP production (***P = 0.001, two-way ANOVA). (B) Measurement of luminescence over time in cells stimulated with either culture medium or 10 μg/mL of CF12 mAb only (without rhFSH). A representative experiment is shown. Results are expressed as relative luminescence units (RLU).

The effect of CF12 mAb on FSH and its receptor was shown by examining cAMP production in HEK293 cells overexpressing hFSH-R stimulated with increasing doses of rhFSH alone (Fig. 2). The dose–response curve exhibited a half-maximal concentration (EC_50_) of 476 ± 122 pM. When increasing doses of rhFSH were pre-mixed with a fixed dose of CF12 mAb (10 μg/mL) before addition to the cells, the dose–response curve shifted to the left, and the EC_50_ of rhFSH was significantly lower (99 ± 29 pM, **P < 0.01 Wilcoxon matched-pairs test). In addition, the extrapolated maximum response (*E*_max_) of rhFSH-induced cAMP was enhanced by 42.1 ± 5.8% in the presence of CF12 mAb (Fig. 2A, ***P = 0.001, two-way ANOVA). As a control, when cells were stimulated with CF12 mAb only (10 μg/mL), no cAMP production was shown, demonstrating that CF12 mAb only stimulates the FSH-R in the presence of rhFSH (Fig. 2B). The cell survival of HEK293 hFSH-R GloSensor cells was assessed using a 3-(4,5-dimethylthiazol-2-yl)-2,5-diphenyltetrazolium bromide (MTT) assay to compare cell activity when stimulated with rhFSH only or in combination with 10 μg/mL CF12 mAb. No difference was observed, demonstrating the absence of toxicity of CF12 mAb on these cells (see Supporting Information Fig. 4).

**Figure 3:**
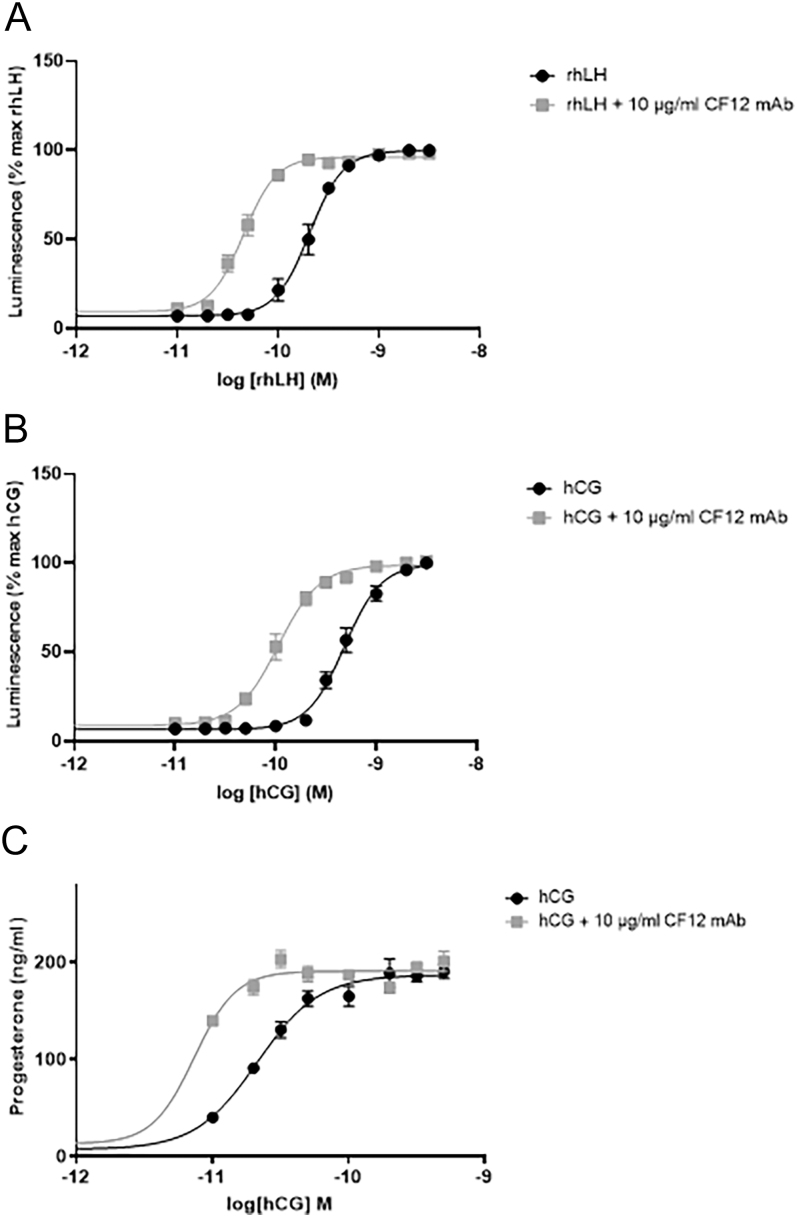
Increase in cAMP activity in mLTC-1 stimulated with either rhLH or hCG ± CF12 mAb at 10 μg/mL. Produced cAMP was quantified using HitHunter® cAMP assay for Biologics and expressed as relative luminescence units (RLU). Graphs represent the mean ± SEM of five independent experiments, each done in duplicate. The addition of CF12 mAb to rhLH (A) and to hCG (B) significantly increased cAMP production (*P = 0.05 and ***P = 0.05 for rhLH and hCG, respectively, two-way ANOVA). (C) Increase in progesterone secretion in mLTC-1 stimulated with increasing doses of hCG ± 10 μg/mL CF12 mAb.

mLTC-1 expressing endogenous LH/CG-R were stimulated with increasing doses of either rhLH (Fig. 3A) or hCG (Fig. 3B). Cells produced cAMP under rhLH and hCG stimulation. When the different doses of rhLH or hCG were pre-mixed with a fixed dose of CF12 mAb (10 μg/mL) before addition to cells, the shift of the dose–response curves occurred as for rhFSH. The EC_50_ of rhLH decreased from 207 ± 36 pM to 45.2 ± 7.1 pM with the addition of CF12 mAb (*P = 0.05), while the EC_50_ of hCG decreased from 589.4 ± 129.2 pM to 120 ± 31 pM (*P = 0.05), clearly demonstrating that CF12 mAb potentiates rhLH/hCG. The potentiating effect was also observed on progesterone secretion by mLTC-1: the EC_50_ of hCG was shifted from 21.1 ± 0.9 pM to 7.2 ± 0.5 pM when in combination with CF12 mAb (Fig. 3C).

Finally, the effect of CF12 mAb on human thyroid-stimulating hormone (hTSH) was investigated in HEK293 cells overexpressing hTSH receptor (hTSHR). The addition of CF12 mAb at 10 and 100 μg/mL to increasing concentrations of hTSH did not change the potency nor the efficacy of hTSH, demonstrating that CF12 mAb does not cross-react with this hormone (see Supporting Information Fig. 5).

### Confirmation of potentiating effect *in vivo* in immature female and male rats

**Figure 4:**
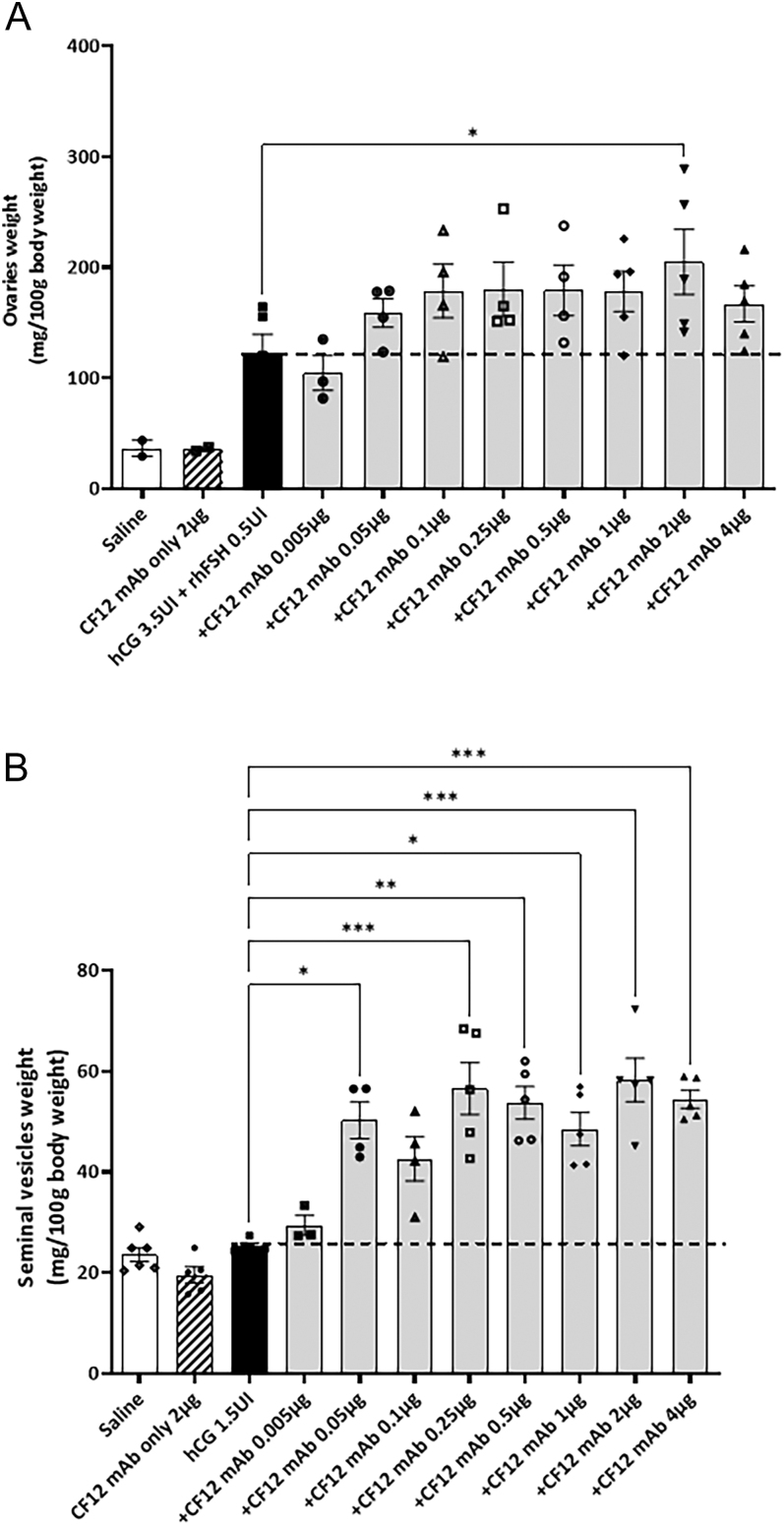
*In vivo* effect of CF12 mAb on rhFSH using Steelman and Pohley bioassay (A), and on hCG using Van Hell bioassay (B). Mean ± SEM, *n* = 5 animals per group. *P < 0.05, **P < 0.01, ***P < 0.001, non-parametric one-way ANOVA (Kruskal–Wallis test, followed by a Dunn’s post-test).

The Steelman and Pohley bioassay (Steelman & Pohley 1953) was undertaken using increasing doses of CF12 mAb pre-mixed with rhFSH and hCG before injection into immature female rats. After 3 days of injections, ovaries were dissected and their weight ratio was expressed per 100 g of body weight (Fig. 4A). Results showed that the effect of CF12 mAb on the ovaries was relatively dose dependent. The maximal response was observed at 2 μg/injection. Although this bioassay is known for its high inter-individual variability, 2 μg/injection induced a significant 1.7-fold increase in ovarian weight (*P < 0.05) even though this experiment was conducted on only five animals. CF12 mAb alone injected at 2 μg/injection did not have any effect on ovarian weight, confirming that CF12 mAb acts as a potentiator only in the presence of FSH.

Similarly, the Van Hell bioassay (Van Hell *et al.* 1964) assessed increasing doses of CF12 mAb pre-mixed with hCG before injection into immature male rats for its ability to potentiate LH/CG. After 4 days of injection, seminal vesicles were dissected and their weight ratio was expressed per 100 g of body weight (Fig. 4B). CF12 mAb effect on weight of seminal vesicles was relatively dose dependent, with a significant response observed as low as 0.25 μg/injection, giving a 2.2-fold increase (**P < 0.01). As shown previously, CF12 mAb injected alone at 2 μg/injection did not have any effect, confirming once more the need for the presence of LH hormone for the expression of CF12 mAb’s potentiating activity.

### Exploration of CF12 mAb effect on spermatogenesis in hypogonadal adult rats

**Figure 5:**
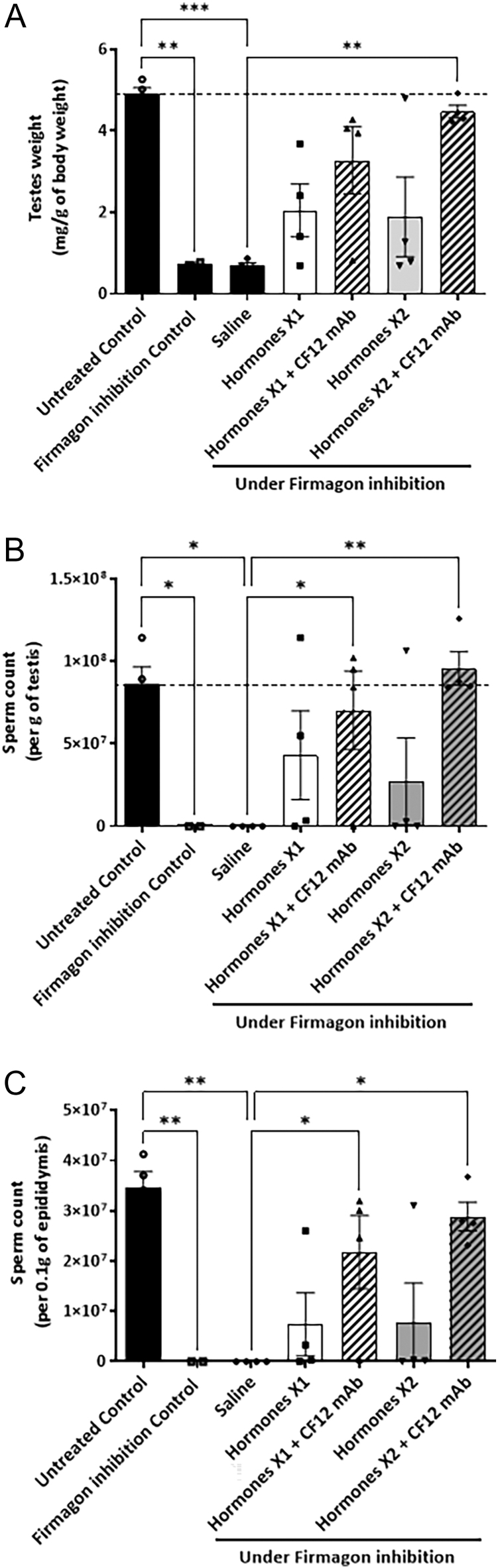
The effect of CF12 mAb on spermatogenesis in azoospermic rats. Wild-type rats were treated with GnRH antagonist to induce azoospermia, and then treated with hormones for 8 weeks at two different concentrations (Hormones X1 and Hormones X2) ± 20 μg/kg CF12 mAb (*n* = 4 rats per group). Significant differences were observed for testes weight (A), sperm count in the testes (B), and sperm count in the epididymides (C). *P < 0.05, **P < 0.01, ***P < 0.001, non-parametric one-way ANOVA (Kruskal–Wallis test) followed by a Dunn’s post-test to compare the different treatment groups.

Inhibition of endogenous gonadotropin secretion in Wistar Han rats with a long-acting gonadotropin-releasing hormone (GnRH) antagonist created an azoospermic adult rat model. Seven weeks after the first injection of the GnRH antagonist, the testes weight of the rats was significantly reduced (Fig. 5A, **P < 0.01). After 8 weeks of treatment, testes weight remained at pre-treatment levels in the group treated with saline and was significantly lower than untreated controls (Fig. 5A, 0.7 ± 0.06 mg of testes weight/g of body weight vs 4.91 ± 0.15 mg/g, ***P < 0.001). When treated with gonadotropins at the first dose (Hormones X1), the testes weight ratio increased to 2.05 ± 0.65 mg/g of body weight. Doubling the dose of gonadotropins (Hormones X2) did not further increase the weight of testes (1.89 ± 0.98 mg/g of body weight). However, the addition of CF12 mAb to the gonadotropin treatment increased the testes weight with Hormones X1 and Hormones X2 by 1.6-fold and 2.4-fold, respectively (3.28 ± 0.82 and 4.48 ± 0.16 mg/g of body weight), revealing that Hormones X2 + CF12 mAb reached significance when compared with saline (**P < 0.01).

Testes weight correlated well with sperm count in the testes (*r* = 0.94) and in the epididymides (*r* = 0.96). Rats treated for 7 weeks with the GnRH antagonist only, or thereafter with saline for 8 weeks, did not have spermatids in the testes nor sperm in the epididymides (Fig. 5B and C). Eight weeks of treatment with gonadotropins started spermatogenesis, but doubling the dose of hormones did not improve the outcome (Fig. 5B, 43.2 × 10^6^ ± 26.9 × 10^6^ and 27.3 × 10^6^ ± 26.4 × 10^6^, respectively, for Hormones X1 and Hormones X2), whereas adding CF12 mAb on top of Hormones X1 or Hormones X2 significantly improved spermatid count in the testes by 1.6- and 3.5-fold, respectively (Fig. 5B, 70.4 × 10^6^ ± 23.8 × 10^6^, *P < 0.05 and 95.8 × 10^6^ ± 10.1 × 10^6^, **P < 0.01). Similarly, sperm count in the epididymides with Hormones X1 or Hormones X2 was not significantly different (Fig. 5C, 7.4 × 10^6^ ± 6.3 × 10^6^ and 7.9 × 10^6^ ± 7.7 × 10^6^, respectively) but was increased by 2.9-fold and 3.7-fold, respectively, when CF12 mAb was added to the hormone regimen (Fig. 5C, 21.7 × 10^6^ ± 7.3 × 10^6^, *P < 0.05 and 28.9 × 10^6^ ± 2.9 × 10^6^, *P < 0.05, respectively, when compared to saline). Spermatid count in the testes in the group treated with Hormones X2 + CF12 mAb was equivalent to untreated control wild-type animals that were not treated with the GnRH antagonist (Fig. 5B).

### Demonstration of CF12 mAb efficacy on ovulation induction in adult ewes

**Figure 6:**
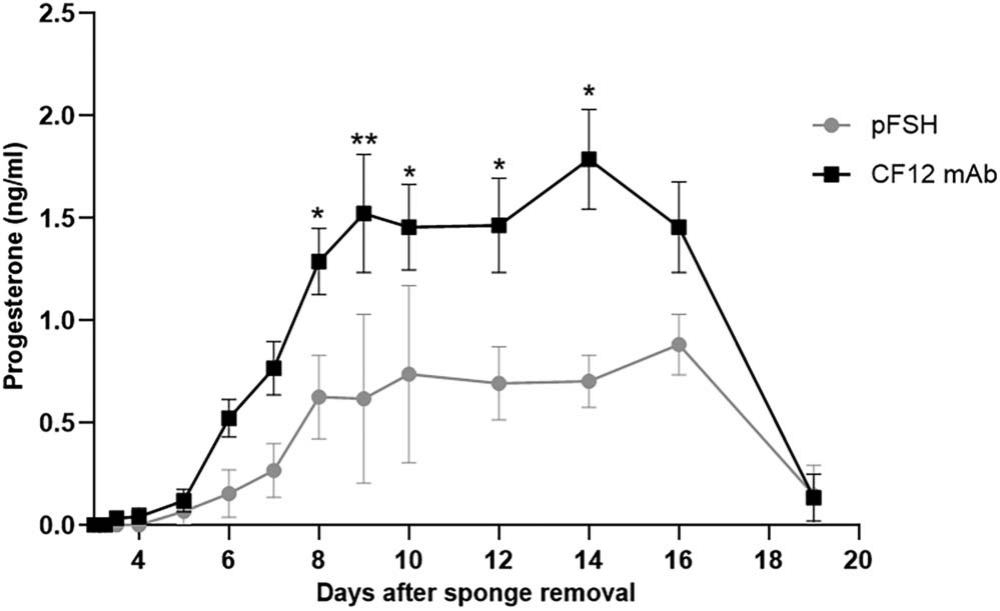
Progesterone secretion in ewes treated with either pFSH or CF12 mAb alone for ovulation induction was measured from the day of progestagen sponge removal until the end of the luteal phase. Females that did not ovulate were excluded from this analysis. Progesterone levels were normalized per corpus luteum and expressed as mean ± SEM. *P < 0.05, **P < 0.01, non-parametric two-way ANOVA, followed by Tukey’s post-test.

Laparoscopy was used to determine that treatment with CF12 mAb alone induced ovulation in all treated ewes (7/7; ovulation rate = 100%), whereas only four out of 11 pFSH-treated females ovulated (ovulation rate = 36%). Ovulations were confirmed by measuring progesterone secretion levels throughout the luteal phase (Fig. 6). Ewes treated with CF12 mAb exhibited significantly higher progesterone secretion compared to those treated with pFSH from day 8 post vaginal sponge removal (0.63 ± 0.29 ng/mL with pFSH and 1.29 ± 0.08 ng/mL with CF12 mAb, *P < 0.05) to day 14 (0.70 ± 0.18 ng/mL with pFSH and 1.79 ± 0.12 ng/mL with CF12 mAb, *P < 0.05), representing a two-fold and 2.56-fold increase, respectively.

## Discussion

In the present series of proof-of-concept experiments, we describe CF12 mAb, a monoclonal murine anti-gonadotropin antibody, and characterize its potentiating effects on FSH, LH, and hCG bioactivities in both female and male animal models.

First, we have shown that CF12 mAb combined with rhFSH enhances *in vitro* FSH-R-mediated intracellular cAMP production, achieving the same effect as rhFSH alone but with a five-fold lower dose of rhFSH, and increasing the maximal effect by 40%. A similar five-fold enhancing effect was observed on LH/CG-R-mediated intracellular cAMP production with rhLH or hCG, although the maximal effect remained unchanged. By contrast, CF12 mAb exhibited no effect on hTSH. The mechanism of action of CF12 mAb is under investigation, but one can speculate that the binding to FSH, LH/CG, and TSH is probably due to the common alpha subunit, whereas the potentiating effect probably involves the beta subunit, as TSH is not potentiated by CF12 mAb.

Then, we confirmed the potentiating effect of CF12 mAb in male and female animal models. We have shown that CF12 mAb increases the stimulating effect of rhFSH combined with hCG by doubling the ovarian weight in immature female rats. Similarly, we have shown that CF12 mAb potentiates the LH activity of hCG, doubling the weight of seminal vesicles in immature male rats. In azoospermic adult male rats treated for 8 weeks (one cycle of spermatogenesis), a gonadotropin cocktail increases the weight of testes and epididymides, as well as sperm count in both, but doubling the hormone doses did not produce any additional effect. Adding CF12 mAb, however, increases the effect of both gonadotropin doses with a greater impact on the highest dose. Importantly, CF12 mAb combined with gonadotropins completely restores spermatogenesis to the level of normal control animals within one cycle of spermatogenesis only.

Across all these *in vitro* and *in vivo* results, CF12 mAb was tested in combination with exogenous hormone. We then confirmed that CF12 mAb potentiates endogenous hormones in the same way by investigating the effect of CF12 mAb injected alone on the induction of ovulation in mature ewes. While a classic pFSH treatment led to ovulation in just 36% of ewes, CF12 mAb alone triggered ovulation in 100% of ewes with concomitant higher levels of circulating progesterone, with no exogenous FSH treatment. Based on the *in vitro* work that confirmed CF12 mAb has no effect alone on FSH-R and LH-R, we can deduce that CF12 mAb enhances the bioactivity of gonadotropins produced naturally by ewes. Our conclusion is therefore that CF12 mAb is able to potentiate the action of gonadotropins, either endogenously produced or administered exogenously.

The induction of ovulation in all females treated with CF12 mAb can be seen as analogous to the induction of ovulation by naturally occurring potentiating anti-eCG Abs found in livestock animals such as ewes and goats treated with eCG. Previous studies have also described potentiating antibodies against polypeptide hormones such as epidermal growth factor (Schechter *et al.* 1979), human insulin-like growth factor-I (Stewart *et al.* 1993), and human growth hormone-releasing hormone (Pell & James 1995), as well as against larger protein hormones such as human growth hormone (Holder *et al.* 1985, 1988, Aston *et al.* 1986), and hTSH (Holder *et al.* 1987).

Beyond this, two studies have described a potentiating effect of anti-FSH Ab *in vivo*. The first described that an mAb was able to enhance the effect of bovine FSH on a uterine growth assay in mice (Glencross *et al.* 1993). The second study described the development of a polyclonal anti-peptide antibody against the ovine FSHβ subunit, which improved the biological activity of exogenous FSH on uterine and ovarian weight gain assays in mice (Ferasin *et al.* 1997). However, their possible effect on ovulation induction has not been demonstrated, as described here for CF12 mAb.

The overlapping roles of FSH and LH have already been described in male animals and humans. LH-R signaling defects deeply impair testosterone production, leading to azoospermia, but strong FSH-R activation can reverse azoospermia and partially restore fertility even though testosterone levels remain minimal (Oduwole *et al.* 2018*a*,*b*). In hypogonadotropic hypogonadal men, FSH is used to improve quantitative and qualitative sperm parameters, while hCG mimicking LH action on their common LH/CG-R is used to increase spermatogenesis (Santi *et al.* 2020, Esteves *et al.* 2024). However, there have been no previous studies that use anti-gonadotropin antibody to potentiate hormone effects in male animal models, showing a reversal of azoospermia and improvements in sperm parameters, as demonstrated here.

Our study of CF12 mAb is therefore the first to show a potentiating effect of an anti-gonadotropin Ab *in vivo* both on the induction of ovulation and enhancing progesterone secretion in females, and on stimulating and restoring spermatogenesis in males. An anti-gonadotropin antibody such as CF12 mAb potentiating both FSH and LH/CG activity could be of interest for therapeutic approaches based on the use of both hormones. While treatments that potentiate both FSH and LH activity may be particularly relevant for women with reduced endogenous LH activity (Leão & Esteves 2014), they might not be the most suitable option for all patients (Gao *et al.* 2021).

### Next steps with CF12 mAb: what might the future hold?

As CF12 mAb was produced in mice by immunization using rhFSH, its wide range of action warrants further investigation of paratopes and epitopes, providing a better understanding of its differential action on each of the three gonadotropins and their two cognate receptors, FSH-R and LH/CG-R.

It is also hoped that our potentiating antibody could represent a significant advance in reproductive medicine, enabling improvements in the treatment of infertility. Infertility affects more than 186 million women and men worldwide, with 17.5% of people experiencing it at some point in their lives (Anon 2023). Current infertility treatments are based on repeated injections of gonadotropins that can be burdensome for women undergoing ART and men being treated for their infertility. An antibody such as CF12 mAb that potentiates the bioactivity of exogenous gonadotropins might allow the use of lower hormone doses and/or less frequent injections. Furthermore, the possibility of stimulating the action of a patient’s own hormones using the antibody alone would broaden the range of infertility care strategies. It is anticipated that the impressive *in vivo* and *in vitro* results presented here could indicate a high rate of successful outcomes in human fertility treatment and also reduce the burden of treatment for patients. If developed, a humanized variant of CF12 mAb that potentiates both endogenous and exogenous gonadotropin activity in females and males may advance the treatment of infertility by increasing the efficacy of hormones.

## Supplementary materials



## Declaration of interest

EK, JD, LD, SC, and MCM are employed by Igyxos Biotherapeutics. MCM is founder of Igyxos Biotherapeutics and a Board of Directors member. PB and RF are members of the Igyxos Biotherapeutics scientific advisory board.

## Funding

This work was funded by Région Centre Val de Loire, AAP PME Innovation, contract #00087445, and by Igyxos Biotherapeutics.

## Author contribution statement

EK, PB, RF, and MCM designed the research. EK, JD, LD, SC, and MCM performed the research. EK and MCM analyzed the data. EK and MCM wrote the paper with the input of all the authors.
