# Supplementary material for: Monoclonal antibody potentiating gonadotropin activity in vitro and in vivo in male and female rats and in ewes
Source: Reproduction. 2025 Sep 15;170(4):e250212. doi: 10.1530/REP-25-0212 (PMC12444799; doi:10.1530/REP-25-0212)

Supporting information

Monoclonal antibody potentiating gonadotrophin activity *in vitro* and *in vivo* in male and female rats and in ewes

Authors

Elodie Kara[1*], Jérémy Decourtye[1], Laurence Dupuy[1], Sophie Casteret[1], Philippe Bouchard[2], René Frydman[2], Marie-Christine Maurel[1]

[1]Igyxos Biotherapeutics, Centre INRAE Val de Loire, Nouzilly, F-37380, France

[2]Hôpital Foch, Service de Gynécologie Obstétrique, 40 Rue Worth, 92150 Suresnes, France

[*]Corresponding author: elodie.kara@igyxos.com

Isotyping of CF12 mAb

The isotype of the mAb produced by the selected clone (CF12) was determined by ELISA using a commercial kit from RD-Biotech (Besançon, France) (Table 1).

Supporting Information Table 1: Isotyping of CF12 mAb.

Specificity	OD_{450}
IgG1	0.089
IgG2a	0.069
IgG2b	0.070
IgG3	0.077
**IgM**	**0.639**
**Kappa**	**0.600**
Lambda	0.118
**H+L**	**0.752**

Cell viability assessment

Cell viability was assessed by a 3-(4,5-dimethylthiazol-2-yl)-2,5-diphenyltetrazolium bromide (MTT) assay using the CellTiter 96® Non-radioactive viability assay (Promega, Madison, WI, USA). HEK293 hFSH-R GloSensor cells were seeded at 20 000 cells/well in 96-well plates and cultured overnight in

100 µl media. On the day of assay, increasing concentrations of rhFSH alone or mixed with 10 µg/ml CF12 mAb were incubated 20 min at 37°C. Cells were then stimulated with 10µl of each mix. Forty-eight hours later, 15 µl of tetrazolium reagent were added in each well and incubated for 4 h. One hundred microliters of solubilization/stop solution were then added per well and the absorbance at 570 nm was recorded 18 h later using an ELISA plate reader.

Supporting Information Fig. 1. Schematic diagram of experimental design on adult male rats

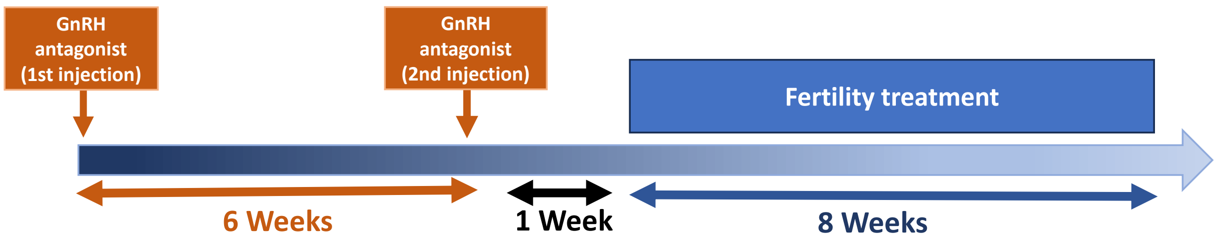

Supporting Information Fig. 2. *In vitro* bioassay in HEK 293 cells expressing FSH-R, showing enhanced intracellular cAMP activity following stimulation with increasing doses of ovine FSH (oFSH), and a shift of the dose-response curve to the left following stimulation with oFSH + CF12 mAb, demonstrating the potentiating effect of CF12 mAb on oFSH *in vitro*.

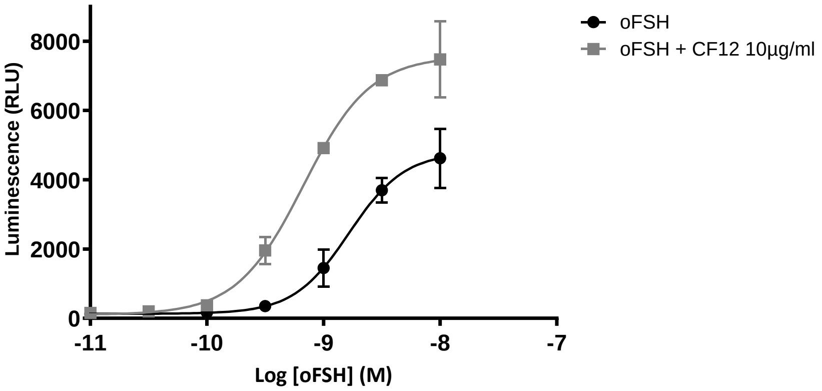

	oFSH	oFSH + CF12 10µg/ml
EC50	1.658e-009	6.729e-010

Supporting Information Fig. 3. Schematic diagram of experimental design on adult ewes

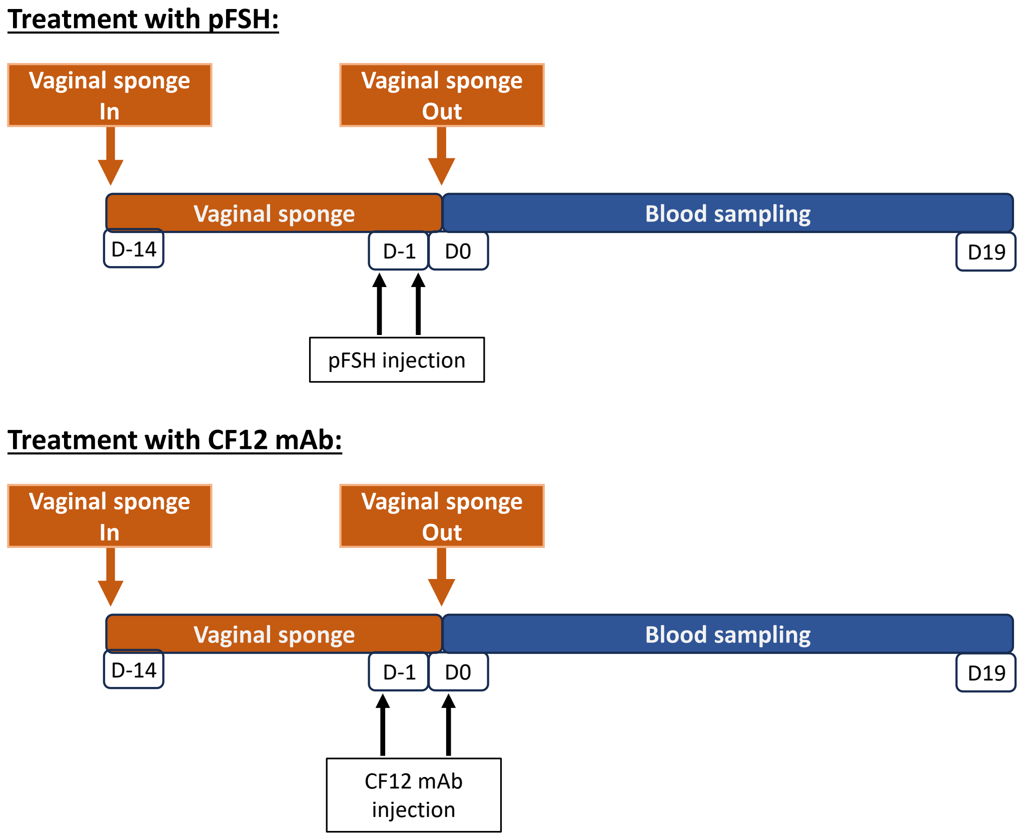

Supporting Information Fig. 4. HEK293 hFSH-R GloSensor cell viability was assessed after stimulation with rhFSH alone or in combination with 10 µg/ml of CF12 mAb. The addition of CF12 mAb at 10 µg/ml to rhFSH did not increase nor decrease cell viability. Results are expressed as a % of basal MTT activity, obtained when cells were stimulated for 48 hours with culture medium only (baseline of rhFSH) or with culture medium + 10 µg/ml CF12 mAb (baseline of rhFSH + 10 µg/ml CF12 mAb).

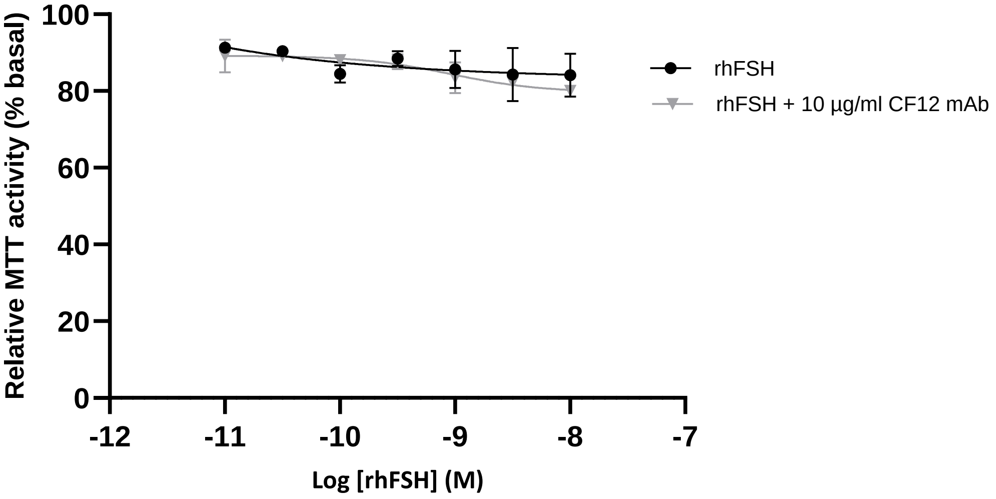

Supporting Information Fig. 5. *In vitro* bioassay in HEK 293 cells expressing TSH-R, showing enhanced intracellular cAMP activity following stimulation with increasing doses of human TSH (hTSH), with no effect of CF12 mAb on hTSH dose-response curve (representative experiment done in duplicate).

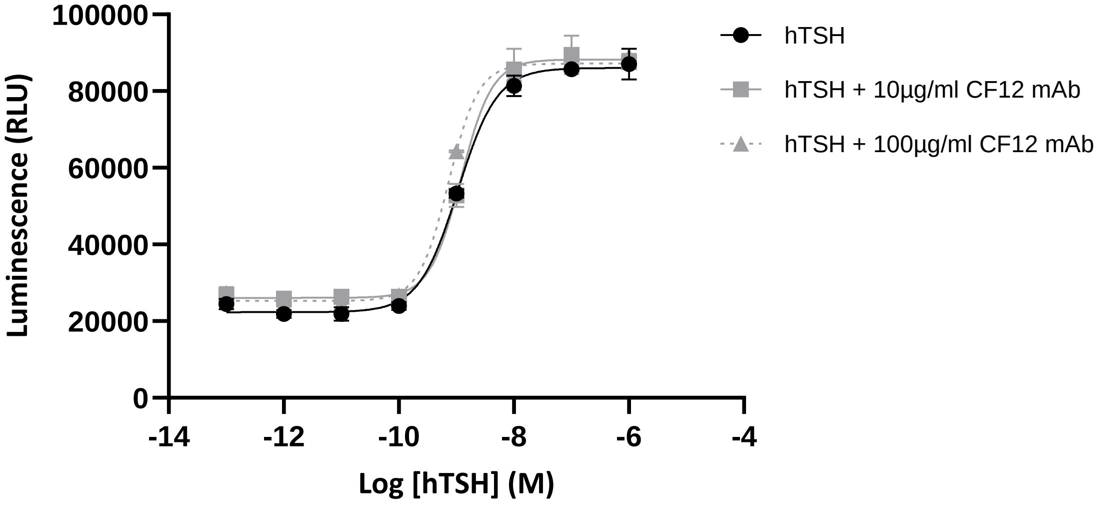

Supplement: Supplementary file 1 [file supplementary_materials.pdf]
